# Assessment of the magnitude and associated factors of immunological failure among adult and adolescent HIV-infected patients in St. Luke and Tulubolo Hospital, Oromia Region, Ethiopia

**DOI:** 10.11604/pamj.2015.21.291.6831

**Published:** 2015-08-20

**Authors:** Bekelech Bayou, Abay Sisay, Abera Kumie

**Affiliations:** 1 ICAP-Ethiopia, Addis Ababa, Ethiopia; 2 Management Science for Health (MSH), Heal TB, Ethiopia; 3 Addis Ababa University, School of Public Health, Addis Ababa, Ethiopia

**Keywords:** HIV/AIDS, magnitude, risk factors, immunological, predictors, treatment failure

## Abstract

**Introduction:**

The use of antiretroviral therapy (ART) has become a standard of care for the treatment of HIV infection. However, cost and resistance to ART are major obstacles for access to treatment especially in resource-limited settings. In this study, we aimed to assess the magnitude and associated factors of Immunological failure among adult and adolescent HIV infected Patients (with age ‘15yrs) on Highly Active Antiretroviral Therapy (HAART) in St. Luke and Tulu Bolo Hospitals, Oromia Region, Ethiopia.

**Methods:**

A retrospective follow-up study was conducted among HIV-infected patients initiated 1st line ART at St. Luke and Tulu Bolo Hospitals, South West Shoa Zone, Oromia, Ethiopia.

**Results:**

A total of 828 patient charts were reviewed. 477(57.6%) were female and the median age was 32 years. The median baseline CD4 count was 148cells/mm3. The most common prescribed ART was TDF based (36.7%). Out of 828 patients chart reviewed 6.8% (56) were developed immunological failure. Out of them only 20 (2.4%) were detected and put on second line regimen. The incidence of immunological failure was 1.8 cases per 100 person years of follow-up. Patients who had not disclosed their HIV status to any one had high risk of immunological failure compared with patients those who had disclosed their HIV status (AHR, 0.429; 95% CI 0.206 - 0.893; P-value=0.024).

**Conclusion:**

Non disclosures of HIV status and with ambulatory of baseline functional status were found to be predictors of immunological failure. Most of the immunological failure cases were not detected early and not switched to second line ARV regimen. So patients with the above risk factors should be considered for a timely switch to second line HAART.

## Introduction

The human immunodeficiency virus (HIV) epidemic continues to be a major challenge to global health. In 2011, an estimated 34 million people were living with human immunodeficiency virus /acquired immunodeficiency syndrome (HIV/AIDS) worldwide; of them 22.9 million were living in Sub-Saharan Africa [1]. According to 2011 Ethiopia Demographic Health survey (EDHS) and 2012 country report the estimated adult prevalence of HIV is 1.5% [2–4]. In Ethiopia considering the magnitude of the problem use of Highly Active Antiretroviral therapy (HAART) started in 2003 as a fee based treatment and then launched free ART in 2005 and by the end of January 2009 an estimated 180,447 clients had been enrolled in ART [5]. According to HEALTH SECTOR DEVELOPMENT PROGRAMME (HSDP) national report the total number of HIV positive people was estimated at 1,216,908 and out of them, 397,818 were eligible for ART, 333,434 ever started and 247,805 currently on ART [6]. According to 2010 Ethiopian national report only 865 adults and 13pediatric patients are on second line regimen which accounts only 0.6%. Immunological failure is defined as: fall of CD4 count to pre-therapy base line, 50% fall from the on treatment peak value, or persistent CD4 levels below 100cells/mm3 [4]. Knowing factors that can help to predict treatment failure will help to identify clients that are at higher risk of treatment failure so as to change regimen for those who already have failed regimen and delay though maximizing follow in those have potential failure. Routine patient follow-up need to be done as part of the routine clinical follow up and need to have a high index of suspension. Studies shows that only a few patients among those who actually had treatment failure were detected at the ART centers. The mean duration of treatment failure on first line ART and the mean duration from detection of treatment failure to switch to second line ART were longer [3]. Different studies from South Africa, Malawi, Ethiopia and Haiti revealed that the prevalence of treatment failure range from 9.8% -15% [6, 3]. There are various risk factors described in different studies and world health Organization (WHO) guidelines [7]. Yet published data on determining of magnitude and associated factors of Immunological failure are limited. Risk factors for Immunological failure have not been studied in this area. So the aim of this study was to assess the magnitude and associated factors of Immunological failure among adult and adolescent HIV infected Patients (with age ≥15yrs) on HAART and to provide information to optimize assessing of Immunological failure among HIV patients on HAART.

## Methods

### Study area and period

The study was conducted in St. Luke and Tulubolo Hospitals, south west Shoa zone, Oromia region St. Luke hospital is located in Woliso town and at distance of 125 Km and Tulubolo hospital is located in Tulubolo town which is 95 km away from Addis Ababa, the capital city of Ethiopia. St. Luke Hospital ever enrolled HIV positive clients/patients to chronic HIV care starting from April 2006-July 2013 who's age ≥15 years is 3478 (F=2134, M=1344) and patients ever started on Highly Active Antiretroviral Therapy (HAART) for the same age and period is 2126 (F=1225, M=901). For Tulubolo Hospital number of patients ever enrolled in HIV chronic care starting from May 2010-July 2013 (the Hospital has started ART service in May 2010) who's age ≥15 years is 218 (F=142, M=76) and patients put on Highly Active Antiretroviral Therapy (HAART) for the same age is 130 (F=82, M=48) and the study period was from April 2006 up to July 2013.

### Study design

Institutional based retrospective follow up study was carried out among HIV-positive patients on HAART for ≥12 months. Hence the chart of HIV positive patients on HAART for ≥12 months between April 2006 and July 2013 were reviewed retrospectively.

### Source population

Source population was all human immune Deficiency Virus (HIV) infected patients with age ≥15yrs who were on first line Antiretroviral Therapy (ART) regimen in St. Luke and Tulu Bolo Hospitals.

### Study population

The study population was all HIV infected patients with age ≥15yrs who started first line ART in St. Luke and Tulu Bolo Hospitals since April 2006-July 2013.

**Sample size determination** Double proportional formula

nj=Zα/2√(1+1/r)P(1-P)+Zβ√P1(1-P1)+P2(1-P2)/r (P1-P2)2

was used to determine the sample size of the study; EPI-info version 3.5.3 window is used with type 1 error 5%, power of 80% and ratio of exposed to unexposed 1:1, and the sample size has been calculated for exposure status in different variables using the following formula. Proportion of exposure status in these variables is taken from previous studies [8, 9]. And the final sample size for the study was 828.

#### Sampling procedure

Two hospitals which are found in south west shoa zone were used. From both hospitals HIV patients on HAART for ≥12 months has been were selected. The calculated final sample size was 828 with zero none response rate. Systematic random sampling is employed after proportional allocation of the sample for both Hospitals.

### Study variables

The dependent variable of the study was immunologic failure. The independent variables include Socio demographic characteristics, Behavioral status and Base line clinical and Laboratory information.

### Data collection methods and instrument used

To ensure consistency of the data electronic data base, registrations and patient charts was used to extract the data. The data were collected by preparing chart abstraction form.

### Data quality control

Before data collection the data collectors and supervisors were get a one day orientation by principal investigator. The data collectors were Hospitals data clerks and the supervisors were Hospitals ART providers. There was a regular supervision to data collectors by the principal investigator and supervisors to maintain the data quality.

### Data analysis

The data was entered to Epi-data 3.1 version and analyzed using SPSS version 20. Descriptive analysis using frequency and summary statistics was conducted to describe the study subject. Immunologic failure distribution within groups in respect to time after initiation of therapy is estimated using Kaplan Meier method and log rank tests. Bivariate Cox regression analysis has been used to determine the association between independent variables and covariates with significant association (p value

### Ethical consideration

The study was approved by an ethical committee in Addis Continental Institute of Public Health. A support letter to the two Hospitals was obtained from the institute before conducting the data collection. The information that was collected by the study were stored in a file and kept confidential.

## Results

**Base line socio demographic and socio clinical character:** from a total of 828 patients data reviewed retrospectively 57.6% (477) of them were female. 61.1% (506) were married and followed by 14.7% (122) widower, 10.4% (86) never married. The median age of the participant at enrollment was 32.00 year (IQR, 27-39yrs) and 45.7% (378) go through primary education (Table 1).

**Table 1:** Baseline socio demographic characteristics of HIV patients on HAART in St.Luke and Tulubolo Hospitals, April 2006-July 2013.(n=828),Ethiopia

Variable	Frequency	Percent
**Age**		
15-24	81	9.8
25-34	377	45.5
35-44	253	30.6
>=45	117	14.1
**Sex**		
Male	351	42.4
Female	477	57.6
**Marital status**		
Never Married	86	10.4
Married	506	61.1
Divorced	44	5.3
Separated	70	8.5
Widowed	122	14.7
**Educational status**		
No Education	198	23.9
Primary	378	45.7
Secondary	214	25.8
Tertiary	38	4.6
**Patient referral**		
From within the facility or hospital	688	83.1
From outside the facility	140	16.9
**Employment status**		
Employed	279	33.7
Unemployed	549	66.3
**Religion**		
Orthodox	530	64.0
Muslim	105	12.7
Protestant	175	21.1
Catholic	12	1.4
^Others	6	0.7
**Condition of spouse**		
Healthy	175	21.1
Chronically ill Dead	221 102	26.7 12.3
Unknown	255	30.8

^ Patients other than mentioned religions

**Base line clinical and laboratory information:** about 82.5% of patients were working in their functional status at a time of enrollment. The commonest documented OI at time of enrollment were herpes Zoster, followed by oral thrush and TB which accounts 22.3%, 15.8% and 13.7% respectively. At base line the median CD4 count was 148cells/mm3 (IQR, 100-200) and the mean Hemoglobin was 12.08g/dl (SD, 1.934). 99.5%(824) were given cotrimoxzole at the time of ART initiation. At base line 33.5%(278) of the participants were on WHO staging 3 and 4. Thirty nine point two percent (325) of participants know their spouse HIV status (Table 2).

**Table 2:** Base line clinical and laboratory information of HIV patients on HAART in St.Luke and Tulu Bolo Hospitals, Ethiopia, April 2006-July 2013 (N=828)

Variable	frequency	Percent
**Weight**		
< 50	461	55.7
≥ 50	367	44.3
**Functional status**		
Working	670	80.9
Ambulatory	133	16.1
Bedridden	25	3.0
**WHO stage**		
Stage 1	281	33.9
Stage 2	316	38.2
Stage 3	183	22.1
Stage 4	48	5.8
**Base line CD4**		
≤ 200cell/mm3	619	74.8
> 200cell/mm3	201	24.3
**Base line CPT**		
Yes	824	99.5
No	4	0.5
**Base line TB**		
Yes	109	13.2
No	719	86.8
**Base line Hgb**		
< 9 g/dl	53	6.4
9-11 g/dl	207	25
> 11 g/dl	568	68.6

**Magnitude of Immunological failure:** a total of 828 patients were followed retrospectively for a median time of 44 months (IQR 32-56) with a minimum of 18 and maximum 97 months follow up from April 2006-July 2013. Out of 828 patients 6.8% (56) developed immunological failure. The incidence of immunological failure was 1.8 cases per 100 person years of follow-up.

**Figure 1:**
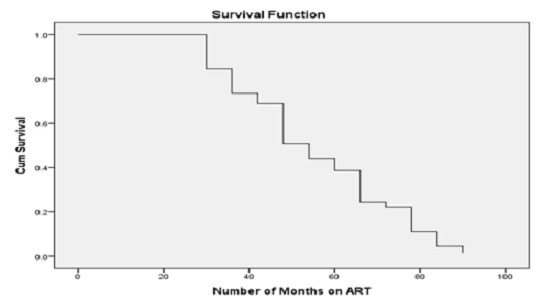
Cumulative probability of not developing immunological failure for patients after initiation of HAART, St.Luke and Tulu Bolo Hospital, April 2006-July 2013

**Time of Immunological failure:** the median time of failure for those qualified immunological failure was 48 months (IQR 35-65 months). Life table showed treatment failure started by 24 month follow-up with 16% and similarly 13%, 6%, 26%, 14%, 12% for the month of 30, 36, 42, 48, and 54 respectively. A sharp drop is seen at 84th month. Life table distribution and the survival function curve is shown in Table 3 and Figure 1. The findings of survival functions between groups were compared using Kaplan-Meier method. To see the significant differences between the groups the Log rank test was used and the result is shown in Table 4, Table 5.

**Table 3:** Life table distribution of probability of immunological failure of patients on HAART St.Luke and Tulu Bolo Hospital, Ethiopia, April 2006-July 2013

*Interval Start Time	Number Entering Interval	Number Withdrawing during Interval	Number Exposed to Risk	Number of Terminal Events	Proportion Terminating	Proportion Surviving	Cumulative Proportion Surviving at End of Interval
0	65	0	65.000	0	0.00	1.00	1.00
6	65	0	65.000	0	0.00	1.00	1.00
12	65	0	65.000	0	0.00	1.00	1.00
18	65	0	65.000	0	0.00	1.00	1.00
24	65	1	64.500	10	0.16	0.84	0.84
30	54	0	54.000	7	0.13	0.87	0.74
36	47	1	46.500	3	0.06	0.94	0.69
42	43	2	42.000	11	0.26	0.74	0.51
48	30	1	29.500	4	0.14	0.86	0.44
54	25	0	25.000	3	0.12	0.88	0.39
60	22	1	21.500	8	0.37	0.63	0.24
66	13	2	12.000	1	0.08	0.92	0.22
72	10	0	10.000	5	0.50	0.50	0.11
78	5	0	5.000	3	0.60	0.40	0.04
84	2	1	1.500	1	0.67	0.33	0.01

Six month interval was taken from the WHO and National guide line which is recommended for immunological follow-up

**Table 4:** Kaplan-Meier and log rank analysis for comparison of survival time for patients on HAART according to base line information, April, 2006-July, 2013. (N=828)

Variable	Mean survival	95% Confidence Interval		
		Lower	Upper	Log rank test
**Sex**				0.083
Male	54.8	48.1	61.6	
Female	47.6	41.3	53.9	
**Age**				0.56
15-24	44.4	33.9	54.8	
25-34	52.9	44.6	61.2	
35-44	51.8	44.3	59.3	
>=45	51.7	40.5	62.9	
**Education**				0.056
NoEducation	47.5	37.8	57.1	
Primary	48.0	41.4	54.7	
Secondary	56.8	47.9	65.6	
Teritiary	77.6	67.5	87.8	
**Marital status**				0.67
Never Married	47.3	34.1	60.5	
Married	52.4	46.3	58.4	
Divorced	61.5	59.4	63.5	
Separated	51.0	44.4	57.5	
Widowed	47.3	32.2	62.3	
**Patient referral**				0.48
From within the facility or hospital	50.6	45.5	55.7	
From outside the facility	63.0	53.1	72.8	

**Table 5:** Kaplan-Meier and Log rank analysis for comparison of survival time for patients on HAART according to base line information, April, 2006-July, 2013. (N=828)

Variable	Mean survival	95% Confidence Interval		Log rank test
		Lower	Upper	
Disclosure status				0.016#
Yes	54.4	48.3	60.5	
No	45.3	39.5	51.1	
Weight at baseline				0.23
< 50	53.3	46.9	59.6	
>=50	49.0	42.1	55.9	
WHO stage at base line				0.3
Stage1	45.4	36.0	54.9	
Stage2	54.2	48.1	60.4	
Stage3	52.9	40.0	65.7	
Stage4	64.5	54.7	74.2	
OI at base line				0.17
Yes	53.9	48.8	59.0	
No	45.6	34.6	56.7	
Base line CD4				0.013#
<=200	54.9	48.9	60.9	
> 200	43.5	37.0	50.0	
Functional status				0.2
Working	49.3	44.2	54.3	
Ambulatory	61.7	50.9	72.4	
Bedridden	50.5	8.3	92.6	
Past TB treatment				0.15
Yes	63.375	50.7	76.0	
No	49.964	45.0	54.8	

# p-value <0.05

**Predictors of Immunological failure:** in bivariate Cox regression CD4 count and disclosure of HIV status were statistically significant. Significance difference was seen for two variables. When survival time for disclosure status was compared, those patients who didn't disclose their HIV status to family member had significantly lower mean survival time, 45.3 with P value=0.016. Significant difference was seen between CD4 count below or equal to 200cell/mm3 and greater than 200cell/mm^3^, time to failure in the latter group was shorter (P value=0.013). No significant association was observed between age, sex, educational status, employment status, marital status, and base line weight of the patient, presence of OI, baseline WHO stage, baseline functional status, presence of past TB and time to Immunological failure. The variable that were found to be significantly associated with immunological failure in bivariate and those with P-value ≤0.2 in Bivariate Cox regression were entered in to multivariate Cox-model. Age and sex was also entered in to the model irrespective of their association to immunological failure as they are the commonest confounders. The multivariate model analysis made evident that disclosure status was significantly associated with immunological failure at P-value less than 0.05 and from the Bivariate Cox regression, functional status was found to be significantly associated with immunological failure. Patients who didn't disclose their HIV status to any one had high risk of developing immunological failure compared with patients those who had disclose their HIV status (AHR, 0.429; 95% CI 0.206 - 0.893; P-value=0.024). Patients with base line functional status ambulatory had risk of developing immunological failure than patients with working and bedridden functional status (HR, 0.337; 95% CI 0.137-0.828; P-value=0.018) (Table 6, Table 7).

**Table 6:** Cox proportional hazard analysis with bivariate and multivariate model for socio demographic, clinical and laboratory, HIV patients on HAART in St.Luke and Tulu Bolo Hospital, Ethiopia, from April 2006-July 2013. (n=828)

Variable	Immunological failure (%)	Immunological success (%)	Crude HR (95% CI)	Adjusted HR#(95% CI)
**Sex**				0.737(0.323-1.683)
Male	30(8.5)	321(91.5)	1	
Female	26(5.5)	451(94.5)	0. 627 (0.365-1.075	
**Age**				
15-24	5(6.2)	76(93.8)	1	
25-34	23(6.1)	354(93.9)	1.847 (0.610-5.591)	
35-44	19(7.5)	234(92.5)	0.913 (0.6416-2.00)	
>=45	9(7.7)	108(92.3)	0.978 (0.440-2.172)	
**Education**				
No Education	12(6.1)	186(93.9)	1	
Primary	30(7.9)	348(92.1)	1.023 (0.543-2.040)	
Secondary	13(6.1)	201(93.9)	0.635 (0.284-1.418)	
Teritiary	1(2.6)	37(97.4)	0.125 (0.016-0.967)	
**Patient referral**				
From within the hospital	50(7.3)	638(92.7)	1	
From outside the facility	6(4.3)	134(95.7)	1.352 (0. 574 -3.180)	

# HR- Hazard Ratio, @ Statistically significant

**Table 7:** Cox proportional hazard analysis with bivariate and multivariate model for socio demographic, clinical and laboratory, HIV patients on HAART in St.Luke and Tulu Bolo Hospital, Ethiopia from April 2006-July 2013. (n=828)

Variable	Immunological failure (%)	Immunological success (%)	Crude HR (95% CI)	Adjusted HR# (95% CI)
**Disclosure status**				
Yes	38(6.8)	517(93.2)	2.056(1.120-3.774)	0.429(0.206-0.893)@
No	18(6.6)	255(93.4)	1	1
**WHO stage**				
Stage 1	17(6.1)	264(93.9)	1	
Stage 2	25(7.9)	291(92.1)	1.916 (0.637-5.761)	
Stage3	10(5.5)	173(94.5)	1.182(0.408-3.426)	
Stage4	4(8.3)	44(91.7)	1.124 (0. 342-3.688)	
**Base line CD4 count (N=820)**				0.517(0.253-1.057)
<=200	38(6.1)	581(93.9)	0.469 (0.252-0.872)	
> 200	16(8)	185(92)	1	
**Functional status**				
Working	43(6.4)	627(93.6)	1	1
Ambulatory	11(8.3)	102(91.7)	0. 557 (0.274-1.130)	0.337(0.137-0.828)@
Bedridden	2(8)	23(92)	1.044 (0 .250-4.354)	0.599(0.111-3.216)
**Weight**				
<50	34(7.4)	427(92.6)	0.718 (0.412-1.253)	
>=50	22(6)	345(94)	1	

# HR- Hazard Ratio

@ Statistically significant

## Discussion

In this retrospective follow-up study Patients on follow-up contributed a total of 3116 person years and incidence of immunological failure was 1.8 per 100 person years of follow-up (PYFUS). From the study found that the independent significant predictors of Immunological failure in patients living with HIV/AIDS after initiation of ART were not disclosing of HIV status to any one of family member and being ambulatory in baseline functional status. As per the WHO and/national ART guide line definition 6.8% of patients had developed immunological failure. The finding is consistent with the finding from India where the prevalence of Immunological failure is 7.3% in participants who had been on HAART for a median of 17 months (IQR:6-30 months) and study conducted in Kenya which shows immunological failure 5.7% [10–13]. The median pre HAART CD4 count observed in this study is 148cell/ mm^3^ which is also comparable with studies conducted in Djibuti with median CD4 count 144cell/mm^3^ and study conducted in Tanzania with median CD4 count 159 cells/mm^3^ (IQR; 65-234) [8, 11, 14]. In bivariate cox regression base line CD4 count ≥ 200/mm^3^ was statistical association in Immunological failure, In contrast studies conducted in India and other sites showed that low Cd4 count (13,8].

### Limitation of the study

This study was conducted retrospectively on Patients chart review. So that most reviewed charts have got missed laboratory tests like platelet counts and pre HAART viral load determination was not included in the study due to absence of Viral load laboratory test in the hospitals.

## Conclusion

According to this study the majority of patients with immunological failure were not diagnosed timely and not switched to second line regimen. So knowing the predictor factors and timing of failure are important parts for care and treatment. Having ambulatory functional status at base line and disclosing of HIV status to family members has identified the independent significant predictors of immunological failure in patients living with HIV/AIDS after initiation of HAART by this study. Based on the finding, the following recommendations were forwarded. Baseline CD4 counts >200cell/mm^3^ should needs further study, sensitization of health care providers to focus on immunological failure and health care providers should be guided to focus on predictors of immunological failure.
